# Health Need Factors Are the Key Drivers of Hospitalization among the Elderly Living Alone: An Analysis of Longitudinal Data

**DOI:** 10.3390/ijerph192215315

**Published:** 2022-11-19

**Authors:** John Rodwell

**Affiliations:** Department of Management & Marketing, Swinburne University of Technology, Hawthorn, VIC 3122, Australia; jrodwell@swin.edu.au

**Keywords:** hospitalization, elderly, living alone, behavioral model, need factors

## Abstract

Elderly people living alone are a large and growing proportion of the population of many developed economies. The elderly, particularly those living alone, are more likely to be hospitalized overnight, with consequent substantial health and financial costs. A widely used model of health service utilization is augmented with social issues that may specifically delineate some of the issues associated with living alone. A longitudinal survey of elderly (aged 65 and over) living alone in Australia with three time points over four years was analyzed using logistic regressions for overnight hospitalization. The main set of data (n = 672) had an average age of 75.91 years and was 70.2% female. The health need factors of self-rated general health and illness severity, along with comorbidity, were the key drivers of hospitalization. There were some individuals with prioritized access to hospitalization due to forms of health funding. The social issues did not independently stand out as drivers of overnight hospitalization, but the complexity of the inter-relationships between issues when studying the health of the elderly began to arise. The results enhance our understanding of health services utilization, within the context of a relatively universal health care system.

## 1. Introduction

Approximately 25.4% of people 65 years of age and over in Australia are living alone [1], likely representing more than 40% of households for that age group. The number of people living alone in Australia is projected to increase by more than 50% over the 25 years until 2041 [2]. In Australia, as in many developed countries, people aged 65 years and over are a notable majority of hospital admissions (e.g., in Sweden [3]), with hospitalization rates more than three times higher than the remainder of the population [4]. Some of the demand from older patients could be avoided, potentially saving health systems substantial amounts of money, and further, older people face an increased risk of hospital-acquired infections and reduced functioning after a hospital stay [5].

Although Australia has a relatively universal health care system, where all residents can access hospital services if needed, those individuals going through the public system may have long waiting times for the scheduling of their procedure, particularly for elective procedures that have been deemed less urgent, and these waiting times may reflect socioeconomic inequalities [6]. Those Australians with appropriate insurance may have access to the private hospital system rather than staying on public system waiting lists in order to have elective procedures sooner before their condition worsens [7].

High hospitalization rates among the elderly emphasizes a need for identifying high-risk people and interventions aimed at reducing their risk [8]. For example, being able to identify those most at risk of hospitalization could inform targeted clinical community care [9]. However, introducing such targeted care models has been met with limited success, possibly because such models often do not consider social considerations and context [9]. Considering the role of more detailed social factors could enhance our understanding of health services utilization [3].

Among the elderly, a key social group at risk of hospitalization are those living alone, where they are 60% more likely to visit an emergency department than those living solely with their spouse [10]. Similarly, elderly people living alone are more likely to be hospitalized than those living with an informal care giver [8], partly because those living alone have higher rates of specific illnesses and accidents such as falls needing treatment [11]. Consequently, this paper investigates social characteristics that may provide a more detailed understanding of health services utilization by including key social characteristics with the widely used (e.g., see [10]) Behavioral Model of health service use [12].

### Augmenting the Behavioral Model of Health Service Use

The Behavioral Model suggests three categories of drivers of health care use: predisposing characteristics, enabling resources, and need [12]. Predisposing characteristics are those that predispose individuals to use health care services (e.g., age and sex). Enabling resources facilitate or impede access to services, such as income and health insurance, and may be a prerequisite for the use of health services (per [13]). However, need is indicated by the individual’s health status, in terms of the individual’s own perceptions, as well as assessments by health professionals [10,12].

The three categories of issues may be interconnected. For example, people on lower incomes, an enabling resource, may be less likely to have preventative screening [8], which may result in more severe health conditions (need) over time. There may also be broader contextual considerations, in that the health care systems of some countries reduce or remove the role of enabling issues (e.g., in Germany, income is not a driver of health care utilization [14]), presumably through the presence of relatively universal health care, widespread health insurance coverage [15], or veteran insurance programs [10].

Across the three categories of drivers within the Behavioral Model, the need factors have often been found to be the stronger driver of health care use (e.g., for hospitalization [10]), suggesting that individuals use these services when medically indicated [13]. However, such a need may be increased when comorbidity is present [8], particularly when comorbidity is present with severe conditions such as chronic obstructive pulmonary disease (COPD) [5]. Conversely, if predisposing characteristics or enabling resources are significant, they may be indicators of inequity of access to health services [12] and a case can be made that achieving equitable health care access entails reducing the impact of predisposing and enabling factors until the main reasons for health care use remaining are need factors [13].

There is a growing set of studies testing the Behavioral Model (e.g., see [14] for a review), and a novel focus here is on the social characteristics of those living alone. Social factors have been suggested to affect hospitalization rates for the elderly beyond specific medical conditions [8]. In terms of mortality, those with stronger social relationships can have a 50% increased likelihood of survival above and beyond the effects of age, sex and initial health status [16]. A similar finding has been found for specific diseases such as COPD, where factors such as material deprivation and poor housing had little effect, but those elderly who were socially isolated were more likely to be hospitalized, although social support did not predict emergency department use [10]. The relationships between social issues may also be overlapping to some degree. For example, social isolation may be associated with physical health and mental health [17], where social issues may be considered as enabling resources [14], but could also be impacting need factors. A possible explanation for these varying degrees of relationship between social issues and hospitalization is the breadth of social issues considered. More complex assessments of social integration have been found to have stronger relationships with health outcomes than simpler measures [16].

Part of the complexity begins with living alone often being considered as a proxy for social isolation, particularly in studies using registry data [18], when that is not necessarily the case. Those who live alone and have few friends or family and have limited contact with people may be socially isolated [19]. However, these social issues may also reflect how social support may impact hospitalization [10]. Clarifying and distinguishing social isolation from loneliness and from social support may help to delineate which social issues have more impact in various situations. More complex consideration of social impacts may reflect the many pathways by which social issues may impact health service use [16].

Social isolation can be considered as a more quantitative assessment of frequency of social contact, which may be a function of network size and diversity, whereas loneliness is more of a subjective assessment of the individual’s expectations and satisfaction with the closeness of contacts [19]. Relatedly, social support is more about the emotional and instrumental aid that may be provided by a social network or community [17]. That is, social support refers more to the real or perceived availability of social resources [16].

Proxies for social support that have been used included marital status, and despite being a somewhat blunt measure, the social support reflected by marital status has been found to be associated with reducing the likelihood of entering a nursing home, although the potential effect of social support on hospitalization has received less attention [20]. Note that while marital status may be associated with certain health outcomes, when psychosocial assessments are included in analyses, the effect of marital status to health outcomes often disappears, suggesting that the protective effects of marriage may occur through socioeconomic and psychological pathways [21]. Social relationships may have either direct or indirect relationships with health, either through direct mechanisms such as biological pathways or indirectly through encouraging healthy behaviors [16]. Clarifying the potential independent effects of these social issues will require them to be tested simultaneously [19].

To better assess the potential effects of social issues on health service utilization for the elderly, this study augments the Behavioral Model [12] with indicators of loneliness, social isolation, and social support. Further, by focusing on those living alone, the social issues may be more clearly delineated, without the potentially confounding effects of marriage. Consequently, this study will analyze the impacts of predisposing characteristics (age, education, marital status), enabling resources (disposable income, health care cards, private health insurance), and need factors (long-term severe illness, comorbidity, body mass index, exercise, smoking, self-rated health, physical functioning, mental health) from the Behavioral Model, along with impacts of loneliness, social isolation, and social support, on overnight hospitalization for the elderly living alone in an Australian health system context.

## 2. Materials and Methods

The sample were drawn from the Household, Income, and Labor Dynamics in Australia (HILDA) dataset, based on a survey intended to be representative of all Australian households [22]. The HILDA Survey has ethics approval number 1647030 from The University of Melbourne. The initial sampling for HILDA was intended to be representative of all Australian households, and the HILDA survey team try to maintain that representativeness across later waves, including a substantial top-up of cases three years before the earliest time period included here [22].

The HILDA survey has a core set of questions asked every year and a rotating set of detailed questions on specific topics that are only asked every four years. That is, the set of detailed health questions are only asked once every four years. The most recent year with the detailed health questions in the data released in early 2022 was 2017 (Time 3) and the previous wave containing detailed health questions was four years earlier in 2013 (Time 1). To avoid possible reverse causality issues that may occur when measures from the same time as the hospitalization period are used (per [23]) the health-oriented predictors were all from previous time periods. Consequently, the detailed health measures are from Time 1, predicting overnight hospitalization at Time 3. More broadly, global measures of health were measured one year prior to the target window at Time 2. The remaining measures of the Behavioral Model were taken as close as possible to the hospitalization window.

The respondents were those who, at Time 3, the interviewer conducting the survey had coded as being a household where the respondent was living alone and were over the age of 64 years. A few cases had shown up as being married and were excluded from the sample. The sample was based on those respondents who had indicated at Time 3 whether or not they had been admitted to hospital for an overnight stay in the last 12 months (Yes/No).

The early indicator of need was assessed at Time 1, four years prior to the hospitalization target window, and assessed serious illnesses, whether acute or chronic, and the presence of comorbidity. The remaining global health measures were assessed at Time 2, one year prior to the target window. The other need factors, as well as predisposing and enabling factors, were assessed at Time 3, along with the social variables. Note that the detailed health measures (e.g., regarding serious illness or hospitalization) are only asked every four years in HILDA. The most recently available dataset with the detailed health questions (Time 3) was in 2017 (with the data used here being released in early 2022). The remaining measures below are listed in approximate time order.

The Time 1 health need data were obtained by asking whether the respondents had been told they had one or more of a list of serious illnesses by a doctor or a nurse, or that they had none of those serious illnesses (0). Respondents indicating that they had diabetes Type 1 or Type 2, blood pressure, arthritis or osteoporosis, asthma, depression, or anxiety were found and classed as being of lower risk of overnight hospitalization (1), whereas those respondents with cancer, heart disease, other mental illness, other circulatory, chronic bronchitis, or emphysema were found to have a higher risk (2) of overnight hospitalization for this older age group. If a respondent had more than one of any of the 11 conditions they were classed as having a comorbidity. The severity dimension and comorbidity dimension were combined because of the notable group they had in common (0, none of these serious illnesses) and the combined variable also enabled the examination of any possible interaction between comorbidity and the severity dimensions. The Hospitalization Severity and Comorbidity variable was coded from (0, none of these serious illnesses), to Low Hospitalization Severity and No Comorbidity (1), to Low Severity with comorbidity (2), High Severity with no comorbidity (3), to High Hospitalization Severity and Comorbidity (4).

Three years later, at Time 2, one year before the hospitalization window, respondents provided their self-rated overall general health, where excellent and very good were combined while good, fair, and poor remained separated. Respondents were also scored for the Physical Functioning and Mental Health sub-scales of the SF36, which was then converted to a 0–100 rating (per [24]). The global health measures were coded to reflect their non-linear relationships with the target variable. The physical functioning scale was grouped as less than 80 versus greater than or equal to 80. The mental health scale was grouped as less than or equal to 81, relative to greater than 81.

The potentially enabling resources associated with loneliness, social support, and social isolation were three separate variables derived from responses at Time 3 to reflect academic definitions such as those of [19]. The loneliness and social support scales were each comprised of four items added together across a seven point ratings, from 1 strongly agree to 7 strongly disagree, and their associated factor analysis checks are detailed in Appendix A. The loneliness scale assessed the extent to which respondents felt lonely and had a Cronbach alpha of 0.765, but had a non-linear relationship with hospitalization and is consequently coded as LTE 20 (lonelier) versus GT20 (less lonely). The social support scale had a Cronbach alpha of 0.813, but was also non-linear and was coded as less than (LT) 16 (higher social support) relative to those scoring greater than or equal to (GTE) 16 (less social support). The social isolation variable was based on an item asking how often you get together socially with friends or relatives not living with you? The frequency of social contact was coded (0) every day or several times a week, (1) about once a week, through to once or twice every three months, and (2) less often than once every three months.

The disposable income was calculated per [22] and initially banded into quintiles, but with the top quintile standing apart, disposable income was coded as the bottom four quintiles relative to the top quintile (beginning at AUD 39,244). The highest level of education received as at Time 3 was grouped as (0) less than or equal to (LTE) grade 12 (high school), or (1) post-school certificate, specifically Certificate 3 or 4, or a Diploma or Advanced Diploma, or (2) Baccalaureate or post-graduate.

Respondents were asked to indicate how often they participated in moderate or intensive physical activity for at least 30 min, where moderate activity would cause a slight increase in breathing and heart rate, such as brisk walking. The responses were grouped such that (1) was not at all and less than once a week, to (2) 1 to 2 times a week, 3 times a week, and more than 3 times a week (but not every day), relative to (3) every day. The body mass index (BMI) of the respondents were grouped per the standard clinical guidelines. The responses regarding whether they smoke cigarettes or any other tobacco products were coded to reflect whether they had ever smoked (1, No, I have never smoked, to 2 No, I no longer smoke, Yes, I smoke daily, Yes, I smoke at least weekly (but not daily), and Yes, I smoke less often than weekly).

At Time 3, respondents indicated their sex (male, female) and age at their last birthday on 30 June 2017 and were coded as either being separated or divorced, relative to a combined group of widowed and never married. In Australia, all residents are covered by Medicare and thus respondents indicated whether, beyond Medicare, they had private health insurance, and if so, what type of health insurance they had. Responses were grouped in terms of whether they had hospital cover (with or without extras) or did not have hospital cover (being those without private health insurance or those who only had insurance for extras). Other enabling resources for overnight hospitalization included respondents with one or more of a set of special health care cards (Veteran’s Treatment Entitlement cards, the Health Care Card, the Pensioner Concession Card or Commonwealth Seniors Health Card), in contrast to those who did not have any of those cards.

## 3. Results

The initial 956 cases matched over the three times across a period of four years had a substantial number of cases with missing values (n = 284) that were missing completely at random (MCAR) per Little’s MCAR test (*p* = 0.194). Of the missing cases, 117 were only missing one value. Only 60 cases (6.3% of 956) appeared to be due to attrition over the four years and may, for an elderly sample, represent moving to nursing homes or death, but these data are not linked to death records, and consequently, the source of attrition is not able to be delineated. The potential impact of the missing cases is analyzed in more detail later in this section.

The descriptive breakdowns of frequencies across the categorical variables are in Table 1. The continuous variable of Age had means (standard deviations) of 77.99 years (7.690) for yes and 75.25 years (7.521) for no, had not spent a night in hospital during the last 12 months. Note that checks of curvilinearity for the continuous variables, following the best practice approach of [25], including checking Box–Tidwell transforms, found that none of the continuous measures had typical curvilinear relationships. Consequently, the more discontinuous non-linear coding was used in the analyses for the relatively continuously-scored variables, except age.

Logistic regression in SPSS 28 was used (per [25,26]) and we derived a model with −2LL = 621.102 that was a significant improvement over the base model (χ^2^ (28) = 118.903, *p* < 0.001), with a Nagelkerke R-square of 0.243. The overall variables that were significant were Severity with Comorbidity (Time 1), self-rated general health (Time 2), the Physical Functioning index (Time 2), being Separated/Divorced (Time 3), having health care cards (Time 3), and Private Health Insurance for Hospitalization (Time 3). The logit parameter estimates for being hospitalized overnight are in Table 2.

Given the large number of cases with missing values, full information maximum likelihood estimations were conducted to generate 20 sets of data replacing the missing values for all variables except the clinically distinct Severity and Comorbidity and the target variable. Logistic regression was then conducted across the imputed datasets and the results are presented on the right-hand side of Table 2. In general, the same variables had the strongest relationships across the two sets of analyses, except that being Separated/Divorced (Time 3) and the Physical Functioning index (Time 2) were not significant in the pooled imputation check analysis. Severity with Comorbidity (Time 1), self-rated general health (Time 2), and having health care cards (Time 3) were significant in both sets of analyses. In the check analysis, Private Health Insurance for Hospitalization (Time 3) had become *p* < 0.10 and Age (Time 3) became *p* < 0.10.

Due to the nature of the changes in results, further checks were conducted, and by swapping out one variable at a time for each of the sets of data, it was able to be determined that Physical Functioning and whether Divorced/Separated or Widowed/Never Married were, together, weakly suppressing the relationship between age and overnight hospitalization.

## 4. Discussion

Some of the variables of the Behavioral Model predicted overnight hospitalization for Australian elderly people living alone. However, as in previous research [10,14], the need factors were the key drivers. That is, across both sets of analyses (original data and the imputed data), illness severity with comorbidity (Time 1) and self-rated general health (Time 2) were the consistent, substantial predictors of overnight hospitalization. These results suggest that individuals were hospitalized overnight because it was medically indicated (in a similar manner to [13]). The impact of comorbidity is more difficult to tease out, with there appearing to be a weak, separate comorbidity effect increasing the likelihood of overnight hospitalization, but the strength of the effect is held back statistically, because, inter alia, of the few respondents with high severity illnesses who did not have comorbidity. If having a severe illness likely to lead to overnight hospitalization, whether with a comorbidity or not, is treated as one group, then there is a weak effect for comorbidity and low hospitalization severity that is consistent across datasets, suggesting that need is increased due to comorbidity, in a similar manner to [8], albeit not as strongly given the high presence of comorbidity among the severely ill. The comorbidity effects may be clearer when focusing on one disease, such as COPD by [10], rather than when studying a general range of diseases.

The variables that were not health indicators (BMI, smoking, exercise) that are often considered as need factors, at least in early versions of the Behavioral Model, were not associated with overnight hospitalization, beyond the stronger need effects such as severity of illness, self-rated health, and comorbidity. Partly because of previous studies finding similar results, later versions of the Behavioral Model (per [12]) began considering BMI, smoking, and exercise more as personal health practices and separated them out from need drivers, a separation that is supported by the results above. The effects of these personal health practices may be more cumulative and preventative, rather than curative, and may need to be investigated in future studies over many time periods.

Of the enabling resources, having some sort of privileged access to hospital fee coverage, whether via some sort of health care card or private hospital insurance, predicted overnight hospitalization. Income did not have an effect, but that may have been a reflection of the low incomes of this sample (elderly living alone) and/or the relatively strong presence of some sort of health care card or private health insurance, which may be considered relatively affordable in the sense that the costs to the individual of private health insurance in Australia are not risk-adjusted. The significant insurance or insurance-like health access enablers in this context may suggest that such discretionary funding may have been used to facilitate “elective” procedures in a context where those without such access may be facing delays in accessing health services for procedures deemed to be less urgent, with potential declines in health due to the worsening debilitation effects of their diseases. Those with the access can instead have the procedures through the private hospital system and avoid delays that may occur via the public system. That is, Australia has (relatively) universal health care—eventually, or if you are critically unwell. These funding enablers may not be the prerequisite for the use of health services suggested by [13], but in this context may represent accelerated health care, where Australians with appropriate insurance may have access to the private hospital system rather than staying on public system waiting lists, thereby also shortening the public hospital waiting lists [7]. This preferential access may be argued to reflect some slight access inequities (per [12]), possibly reflecting socioeconomic inequalities [6].

The argument that equitable health care access entails that predisposing factors and enabling resources should lessen until the main drivers for using health services are need factors [14] is generally borne out here, with the main drivers of health service utilization being need factors. However, there seem to be adjustments or considerations for the speed of what the current policies in Australia see as less urgent hospitalization, where the equity of access is focused more tightly on urgent hospitalization, with less urgent issues requiring hospitalization possibly considered somewhat discretionary and only being available on a less equitable basis.

The predisposing factors investigated in this study were not consistently clear for any one variable, but appear to reflect a set of inter-connected effects. In the initial analyses, being divorced had a lower rate of hospitalization than being widowed or single, but was not significant in the analyses with imputed data. The need factor of physical functioning was also significant in the initial analyses, but was not significant in the analyses with imputed data. Checks across both sets of data, with variables held out one, two, or three at a time, found that physical functioning and being divorced were suppressing the weak relationship between age and overnight hospitalization. Conversely, the suppression effect is that the age variable started strengthening in the pooled analyses. Age may have been prevented from becoming significant because of some range restriction, with an age range of 65 to 100 years, but that would be unlikely to be much of an effect and would have shown up as a non-linear effect in the linearity checks. Another possibility is that as age increases and physical functioning decreases, while more people become widowed, and more of the target group may have gone to nursing homes, who were not included in the sample, but their departure may impact the results here as they went on their own health journey.

Social issues may be considered to be enabling resources [14], although they may also be related to need factors over time. When considered in the same window as the assessment of overnight hospitalization none of the social issues were significant, other than the blunter social assessment embedded in being separated/divorced relative to being widowed/never married, which was significant initially closely interrelated with physical functioning and age. The complexity of the inter-relationships among the behavioral model variables and with the social variables is also reflected in how the social support of marital status had been previously found to reduce the likelihood of going to a nursing home [20]. Unlike with [21], in the above initial analyses the effect of marital status was significant even with the inclusion of economic (disposable income) and psychological variables (e.g., loneliness, social isolation). Again, it is possible that in this case with a focus on overnight hospitalization, rather than a focus on depression [21], the importance of medical necessity was the key underlying issue. Similarly, the overlapping relationships of the social and health issues, such as where social isolation was associated with physical and mental health [17], along with the results above, may suggest that the social issues have important indirect effects. That is, the social variables may have more of an effect indirectly and cumulatively over time. Further, the social issues may have more of a relationship with other health service utilization outcomes than overnight hospitalization.

The possible limitations of the results of this study are particularly about the nature of the national health policy context of this Australian sample. Some example impacts of the context being the relatively universal health care system of Australia have been discussed above. The impact of having private health insurance or some other access to health care funding did have a relationship with overnight hospitalization, despite this relatively universal health care context. Overall, however, this study presents an extra context for consideration in assessing the pattern of results across contexts, particularly given that (per [14]) the majority of longitudinal behavioral model studies were in the context of Germany or the United States of America. There may also have been range restriction issues on some of the variables because the sample were those who live alone, yet there was still a substantial range of scores for each of the social issues, and this sub-group of the population, particularly among the elderly, is a sub-group that warrants attention, not least because of their higher rates of health service utilization. A further limitation may be that the sample analyzed may have a slight bias due to the exclusion of those elderly who had moved to nursing homes or died or moved over the four years and were unable to be contacted (together representing up to 6.3% of the sample), possibly reflecting poorer health or mixed socioeconomic effects. That is, the remaining sample at Time 3 may have been slightly healthier than the whole Time 1 sample if they had been able to be considered at Time 3. Note, however, that movements to nursing homes is a specific topic in the field and, if combined with the effects above, may give a more fulsome picture of the health trajectories of the elderly.

## 5. Conclusions

The need factors of the Behavioral Model were the key drivers of hospitalization among these elderly living alone in Australia. There were some individuals with prioritized access to hospitalization due to health insurance funding of some sort. The social issues did not independently stand out as drivers of overnight hospitalization, but may have suggested the complexity of the inter-relationships between issues when studying the health of the elderly. Together, these results enhance our understanding of health services utilization, particularly within a relatively universal health care system.

Interventions to help the elderly living alone could be aimed at those at high risk of hospitalization by informing targeted community care where, applying [10], programs could evaluate serious illness, comorbidity, and general health, along with changes in marital status and physical functioning, and then be designed to address medical, psychosocial, and functional issues linked to a treatment plan with follow-up. The effectiveness of such interventions could then also be assessed in terms of their impact on hospitalization. Reducing the relative use of overnight hospitalization could generate substantial health and monetary benefits, resulting in an improved quality of life for those over 65 years of age and living alone—a growing proportion of the population in many developed economies.

## Figures and Tables

**Table 1:** The number of respondents for each of the categories used in the analyses by overnight hospitalization at Time 3.

	Hospitalized Overnight in Last 12 Months (Time 3), n = 672	
Categorical Variables	Yes (n = 161)	No (n = 511)
Hospitalization Severity with Comorbidity (Time 1)		
No Severe Illness	10.6%	24.1%
Low Hospitalization Severity and no comorbidity	19.3%	28.6%
Low Severity with Comorbidity	24.2%	27.2%
High Severity with no comorbidity	4.3%	2.3%
High Hospitalization Severity with Comorbidity	41.6%	17.8%
Self-rated overall general health (Time 2)		
Excellent and very good	13.7%	32.7%
Good	33.5%	39.1%
Fair	35.4%	25.2%
Poor	17.4%	2.9%
Physical Functioning (Time 2)		
LT80	80.7%	56.2%
GTE80	19.3%	43.8%
Mental Health (Time 2)		
LTE80	65.8%	49.9%
GT80	34.2%	50.1%
Loneliness (Time 3)		
LTE20, Less lonely	87.6%	93.3%
GT20, More lonely	12.4%	6.7%
Social Support (Time 3)		
LT16 Less social support	9.3%	6.8%
GTE16, More social support	90.7%	93.2%
Social Isolation (Time 3)		
Daily-Several times/week	37.3%	41.3%
1/week to 1–2/3 months	54.3%	55.8%
Less than once every 3 months	9.3%	2.9%
Disposable Income (Time 3)		
Lowest 4 quintiles	85.1%	73.4%
Highest quintile	14.9%	26.6%
Education (Time 3)		
LTE Yr12	59.0%	54.6%
Certificate 3, 4, or Diploma	29.2%	26.6%
Baccalaureate or PG	11.8%	18.8%
Moderate Exercise (Time 3)		
Not at all to <1/week	53.4%	32.5%
1–2/week–3/week	39.1%	54.6%
>3 times per week	7.5%	12.9%
BMI (Time 3)		
<18.5 Underweight	3.1%	2.5%
18.5–<25 Healthy Weight	37.9%	35.2%
25–<30 Overweight	33.5%	33.7%
30–<40 Obese	23.0%	25.4%
GTE40 Very Obese	2.5%	3.1%
Ever Smoked (Time 3)		
Never Smoked	50.3%	53.2%
No longer smoke/smoke daily	49.7%	46.8%
Sex (Time 3)		
Male	30.4%	29.5%
Female	69.6%	70.5%
Single Status (Time 3)		
Separated/Divorced	29.8%	39.1%
Widowed and Never Married	70.2%	60.9%
Health care cards (Time 3)		
No HC card	4.3%	16.0%
Yes, have one or more of those cards	95.7%	84.0%
PHI (Time 3)		
Hospital only or Hospital with Extras	47.8%	49.9%
No PHI or PHI for Extras only	52.2%	50.1%

Note: GTE = Greater than or equal to, LTE = Less than or equal to, GT = Greater Than, and LT = Less than.

**Table 2:** The logistic regression results on having overnight hospitalization at Time 3.

[Ref.: Not Hospitalized]	Hospitalized Overnight in Last 12 Months (Time 3), n = 672			Hospitalized Overnight in Last 12 Months (Time 3), Imputed Pool		
Variables	B (S.E.)	Odds Ratio	95% C.I.	B (S.E.)	Odds Ratio	95% C.I.
Hospitalization Severity with Comorbidity (Time 1)						
No Severe Illness	−0.812 (0.356)	0.444 *	0.221–0.891	−0.499 (0.273)	0.607 †	0.356–1.037
Low Hospitalization Severity and No Comorbidity	−0.538 (0.291)	0.584 †	0.330–1.033	−0.439 (0.226)	0.644 †	0.413–1.004
Low Severity with comorbidity	−0.667 (0.272)	0.513 **	0.301–0.874	−0.661 (0.216)	0.516 **	0.338–0.789
High Severity with no comorbidity	0.779 (0.575)	2.178	0.706–6.720	0.569 (0.462)	1.766	0.715–4.364
High Hospitalization Severity and Comorbidity	0			0		
Self-rated general health						
Excellent and very good	−1.992 (0.487)	0.136 **	0.052–0.355	−1.361 (0.455)	0.256 **	0.104–0.630
Good	−1.581 (0.407)	0.206 **	0.093–0.457	−1.122 (0.373)	0.326 **	0.156–0.680
Fair	−1.539 (0.403)	0.215 **	0.097–0.473	−0.960 (0.367)	0.383 *	0.186–0.790
Poor	0			0		
Physical Functioning (Time 2)						
LT80	0.623 (0.290)	1.864 *	1.056–3.291	0.384 (0.247)	1.468	0.904–2.383
GTE80	0			0		
Mental Health (Time 2)						
LTE80	0.309 (0.229)	1.362	0.870–2.133	0.171 (0.194)	1.187	0.811–1.737
GT80	0			0		
Loneliness						
LTE20 [Less lonely]	−0.260 (0.352)	0.771	0.387–1.535	−0.103 (0.296)	0.902	0.503–1.618
GT20 [More lonely]	0			0		
Social Support						
LT16 [Lower social support]	−0.092 (0.401)	0.913	0.416–2.001	0.099 (0.272)	1.104	0.645–1.891
GTE16 [Higher social support]	0			0		
Social Isolation						
Daily/several times/week	−0.510 (0.483)	0.600	0.233–1.547	−0.330 (0.391)	0.719	0.333–1.554
1/week to 1–2/3 months	−0.716 (0.465)	0.489	0.196–1.216	−0.467 (0.370)	0.627	0.303–1.298
Less than once every 3 months	0			0		
Disposable Income						
Lowest 4 quintiles	0.196 (0.294)	1.217	0.685 −2.163	0.040 (0.242)	1.041	0.648–1.672
Highest quintile	0			0		
Education						
LTE Yr12	0.400 (0.324)	1.491	0.791–2.812	0.301 (0.263)	1.351	0.807–2.263
Certificate 3, 4, or Diploma	0.559 (0.352)	1.749	0.877–3.489	0.374 (0.289)	1.453	0.825–2.561
Baccalaureate and PG	0			0		
Moderate Exercise						
Not at all–<1/week	0.111 (0.395)	1.118	0.515–2.424	0.570 (0.379)	1.768	0.838–3.730
1–2/week–3/week	−0.026 (0.375)	0.975	0.467–2.034	0.313 (0.365)	1.367	0.667–2.804
>3 times per week	0			0		
BMI						
<18.5 Underweight	0.183 (0.864)	1.201	0.221–6.525	0.103 (0.644)	1.109	0.313–3.924
18.5–<25 Healthy Weight	0.652 (0.655)	1.919	0.531–6.929	0.068 (0.510)	1.070	0.394–2.908
25–<30 Overweight	0.292 (0.650)	1.339	0.375–4.782	−0.307 (0.514)	0.735	0.268–2.017
30–<40 Obese	−0.032 (0.655)	0.969	0.269–3.494	−0.307 (0.507)	0.736	0.272–1.987
GTE40 Very Obese	0			0		
Ever Smoked						
Never Smoked	−0.134 (0.210)	0.874	0.579–1.320	−0.223 (0.175)	0.800	0.567–1.129
No longer smoke/smoke daily	0			0		
Sex (Time 3)						
Male	0.060 (0.240)	1.062	0.663–1.701	0.192 (0.187)	1.212	0.840–1.749
Female	0			0		
Single Status (Time 3)						
Separated/Divorced	−0.551 (0.247)	0.577 *	0.355–0.936	−0.242 (0.195)	0.785	0.536–1.151
Widowed and Never Married	0			0		
Health care cards (Time 3)						
No, do not have any of these health care cards	−1.032 (0.470)	0.356 *	0.142–0.895	−0.799 (0.344)	0.450 *	0.229–0.883
Yes, have one or more of those cards	0			0		
PHI (Time 3)						
Hospital only or Hospital with Extras	−0.523 (0.214)	0.593 *	0.390–0.902	−0.301 (0.170)	0.740 †	0.531–1.032
No PHI or PHI for Extras only	0			0		
Age	0.013 (0.015)	1.013	0.984–1.043	0.019 (0.012)	1.020 †	0.996–1.043
Constant	−0.318 (1.509)			−1.257 (1.252)		

Note: † < 0.10, * < 0.05, ** < 0.01. S.E. = Standard Error, C.I. = Confidence Interval, GTE = Greater than or equal to, LTE = Less than or equal to, GT = Greater than, and LT = Less than. The reference categories were set to 0.

## Data Availability

The HILDA data is available and managed by the Melbourne Institute of Applied Economic and Social Research at The University of Melbourne (https://melbourneinstitute.unimelb.edu.au/hilda). The version used above was release 21.

## Appendix A

Those who live alone are often assumed to be socially isolated and/or lonely. In this study, among those living alone, the degree of loneliness, social isolation or social support will be explicitly assessed. In particular, social isolation and loneliness are distinct constructs. Loneliness is more a subjective evaluation of one’s expectations of and assessments about the amount and closeness of contacts, whereas social isolation is more a reflection of frequency of contact within one’s social network [19].

Given the need for specific measures of loneliness, social support and social isolation for the issues investigated in this study, further analyses were conducted to ascertain the nature of their inter-relationships and the discriminant and construct validity of those variables. Initial principal components, then principal axis factor analyses across all 10 relevant items in HILDA found two factors, but had two items that were cross-loaded, that is, loading similarly across the two factors. Checks with and without the cross-loading items led to a clearer two factor solution. The two items that were excluded were: ‘I seem to have a lot of friends’ and ‘I often need help from other people but can’t get it’. The oblique (in SPSS, oblimin) principal axis solution’s pattern matrix is in Table A1.

**Table A1:** Pattern matrix loadings for the two principal axis factors across the eight loneliness and social support items.

Question Item	Factor 1 Social Support	Factor 2 Loneliness
When something’s on my mind, just talking with the people I know can make me feel better	0.862	0.073
I enjoy the time I spend with the people who are important to me	0.801	0.091
When I need someone to help me out, I can usually find someone	0.738	−0.177
There is someone who can always cheer me up when I’m down	0.516	−0.084
I have no one to lean on in times of trouble	0.031	0.789
I don’t have anyone that I can confide in	−0.053	0.768
I often feel very lonely	−0.019	0.583
People don’t come to visit me as often as I would like	0.012	0.549

The two factors above were correlated at r = −0.428 (*p* < 0.001). Note that although that is a high correlation, it does not appear to be high enough to warrant collapsing the factors to one factor. Additionally, recall from the method section above that Loneliness had a Cronbach alpha of 0.765 and Social Support had a Cronbach alpha of 0.813. Further checks were conducted by creating the scales and then comparing the two scales to the Social Isolation item.

The Social Isolation item had a relatively objective focus on frequency of contact and asked: ‘In general, about how often do you get together socially with friends or relatives not living with you?’ Responses ranged from ‘Every day’, ‘Several times a week’, ‘About once a week’, ‘2 or 3 times a month’, ‘About once a month’, ‘Once or twice every 3 months’, to ‘less often than once every 3 months’. Due to the ordinal scaling of the responses to the Social Isolation item non-parametric correlations are also included in Table A2.

**Table A2:** The parametric correlations between the social variables and the non-parametric correlations with Social Isolation.

	Loneliness	Social Support	Social Isolation ^1^
Loneliness	--		0.231/0.299
Social Support	−0.398	--	−0.199/−0.251
Social Isolation	0.282	−0.230	--/--

^1^ The correlations in the right-most column are Kendall’s tau-b/Spearman’s rho, the remaining correlations are all Pearson correlations.

All of the correlations are in the expected directions and although all of the correlations are significant (at *p* < 0.001), partly due to the relatively large sample size, along with the size of the relationships, the correlations are not large and are not at risk of representing collinearity. All of the scales and items are weakly related, but are distinct from each other, further supporting their consideration as separate constructs.
